# Neurons in the pigeon visual network discriminate between faces, scrambled faces, and sine grating images

**DOI:** 10.1038/s41598-021-04559-z

**Published:** 2022-01-12

**Authors:** William Clark, Matthew Chilcott, Amir Azizi, Roland Pusch, Kate Perry, Michael Colombo

**Affiliations:** 1 Department of Psychology, University of Otago, Dunedin, New Zealand; 2 Department of Physics, University of Otago, Dunedin, New Zealand; 3 Department of Systems Biology, Agricultural Biotechnology Research Institute of Iran (ABRII), Karaj, Iran; 4 Department of Biopsychology, Institute of Cognitive Neuroscience, Faculty of Psychology, Ruhr University Bochum, Bochum, Germany

**Keywords:** Neuroscience, Physiology

## Abstract

Discriminating between object categories (e.g., conspecifics, food, potential predators) is a critical function of the primate and bird visual systems. We examined whether a similar hierarchical organization in the ventral stream that operates for processing faces in monkeys also exists in the avian visual system. We performed electrophysiological recordings from the pigeon Wulst of the thalamofugal pathway, in addition to the entopallium (ENTO) and mesopallium ventrolaterale (MVL) of the tectofugal pathway, while pigeons viewed images of faces, scrambled controls, and sine gratings. A greater proportion of MVL neurons fired to the stimuli, and linear discriminant analysis revealed that the population response of MVL neurons distinguished between the stimuli with greater capacity than ENTO and Wulst neurons. While MVL neurons displayed the greatest response selectivity, in contrast to the primate system no neurons were strongly face-selective and some responded best to the scrambled images. These findings suggest that MVL is primarily involved in processing the local features of images, much like the early visual cortex.

## Introduction

The ability to recognise visual objects belonging to different categories is the foundation for object-dependent behaviour across the animal kingdom. The structures that mediate object recognition are most well understood in the primate brain. Ascending visual information from the retina is progressively transformed into a more readable form at each stage of the primate ventral stream, with increasingly complex and viewpoint-invariant representations of faces and other objects emerging at the level of inferior temporal (IT) cortex^1–3^. The discovery of a general purpose circuitry underlying face perception in IT cortex raises the question of whether similar networks have evolved in the visual systems of organisms distantly related to primates.

Similar to mammals, birds have two ascending visual pathways. The thalamofugal pathway is composed of the thalamic dorsolateral geniculate nucleus, which projects to the visual Wulst^4^. The Wulst is possibly homologous with the mammalian primary visual cortex, and forms a retinotopic map of the visual field^5–7^. The visual Wulst is heavily involved in visually guided behaviour, and may participate mainly in pattern vision for small and distant targets in the fovea of the lateral visual field^8–10^. The tectofugal pathway consists of the midbrain visual tectum and its projections via the thalamic nucleus rotundus to the pallial entopallium (ENTO)^11^. ENTO is possibly analogous to parts of extrastriate cortex^12, 13^, containing neurons with large receptive fields well suited for object identification over large areas of the visual field^14^. The entire anterior–posterior extent of ENTO forms a topographic and reciprocal connection with the above-positioned layers of the nidopallium and the mesopallium^15^. The mesopallium ventrolaterale (MVL) is one of the mesopallial visual nuclei of the dorsal ventricular ridge (DVR) in the avian brain, and receives input from both the ENTO as well the intermediate nidopallial layers^13,16^. The tectofugal pathway is thought to be primarily involved in identification of objects in the area dorsalis (a second fovea region) of the frontal visual field^17, 18^.

Neurons in the pigeon visual association regions discriminate between basic stimulus parameters such as pattern, color, amplitude, and spatial frequency^19^. A recent study using linear discriminant analysis (LDA)^20^ also demonstrated that a small population of MVL neurons can discriminate between the features of animate and inanimate objects with greater capacity than at the level of ENTO. The static features of the avian face-region holds ethological relevance for pigeons^21–23^, suggesting that neural specialization related to social aspects of vision may exist in the avian brain. Face-selectivity at the single-cell level outside of primates has only been confirmed in sheep^24^. The purpose of the current study was to assess whether neurons in the pigeon visual system might show selectivity for faces, despite the evolutionary separation and differences in brain organisation from mammals.

## Methods

### Subjects

Fourteen experimentally naive pigeons (*Columba livia*) served as subjects and were housed individually in wire mesh cages in a colony room maintained at 20 °C. The birds had ad libitum access to grit and water, and were fed a blend of wheat, peas, and corn. The pigeons were maintained at 85% of their free feeding weight during the experiment. All experimental procedures were approved by the University of Otago Animal Ethics Committee and conducted in accordance with the University of Otago’s Code of Ethical Conduct for the Manipulation of Animals and the ARRIVE guidelines for the care and use of laboratory animals.

### Apparatus

The equipment was similar to that used in Clark et al*.*^25^. Training and testing of the pigeons was performed using standard operant chambers with dimensions of 32.5 cm (length), 36 cm (width) and 34.5 cm (height). A 17-inch screen (resolution: 1284 × 1024) was used to present stimuli. A Carroll Touch infrared touch frame (EloTouch, baud rate 9600, transmission time 20 ms) was placed directly in front of the screen and registered the XY coordinates of pecks. A transparent plexiglass panel with a single square response key (2.5 × 2.5 cm) was also situated in front of the screen and prevented accidental responses from the pigeon’s body from being registered. Grain reward was delivered via a food hopper 20 cm below the square response key, and was illuminated when raised.

### Stimuli

**Figure 1:**
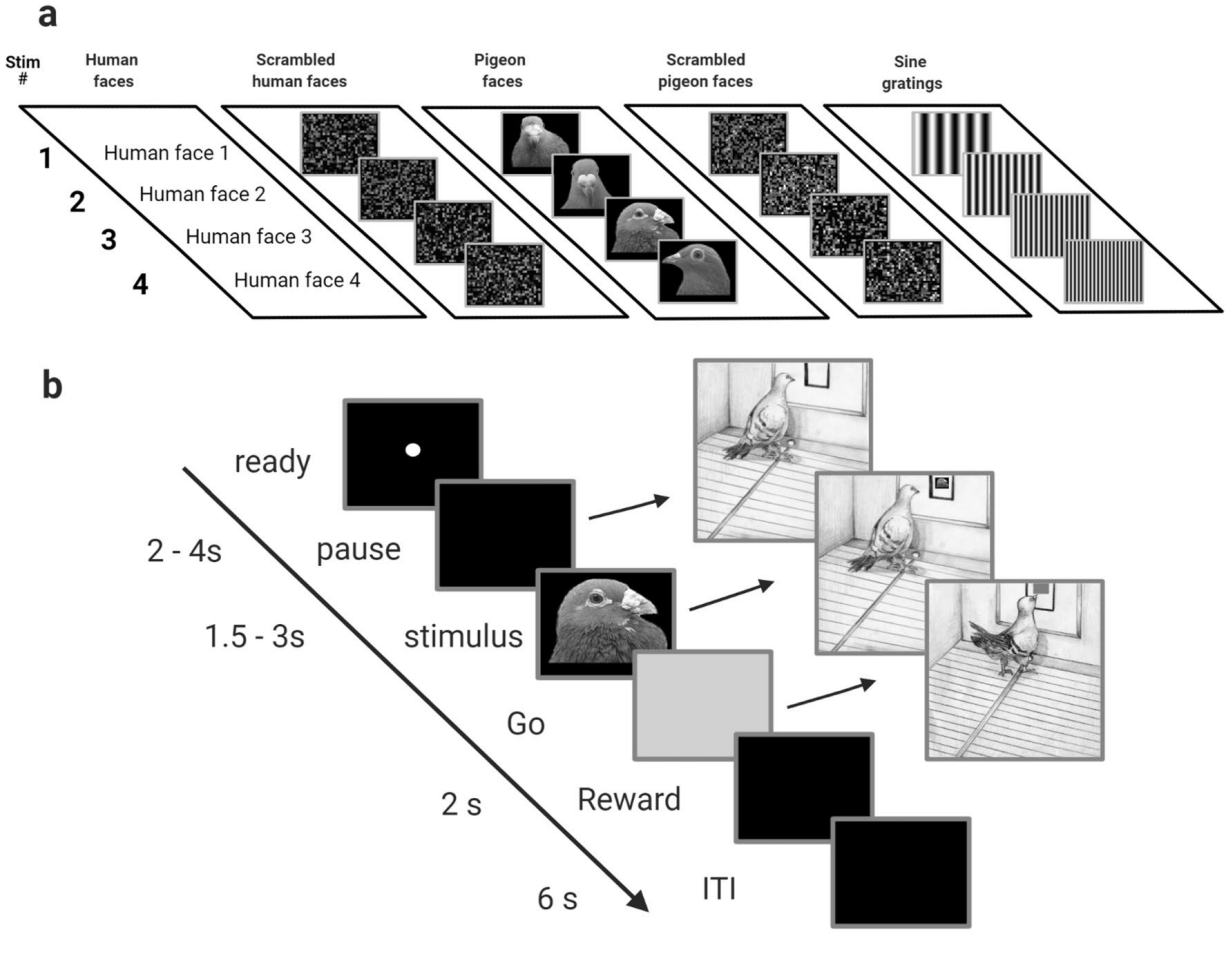
The visual stimuli and behavioural task. (**a**) Images of the five stimulus groupings with the four examples of each grouping used in the experiment. The stimulus groupings consisted of human faces, scrambled human faces, pigeon faces, scrambled pigeon faces, and sine gratings. (**b**) Sequence of events within a single experimental trial. Trials began with an orienting stimulus on screen (white dot) during the ready period. The pigeon pecked the orienting stimulus two times to start a pause period of random duration (2–4 s) with a black screen. Pecks during the pause period extended the duration of the period by 2 s. A stimulus was then displayed for a random duration (1.5–3 s) during the stimulus period. Any pecks during the stimulus period initiated a repeat of the trial from the start of the ITI period. Following the stimulus period, a Go cue (grey square) replaced the stimulus. A peck to the Go cue delivered grain reward from the hopper (2 s) that was paired with a tone (1000 Hz) and illumination of the hopper, followed by a black screen during the 6 s ITI. Pecks during the ITI period extended the duration of the period by 2 s. Scrambled images were created using open access Webmorph software (https://fei.edu.br/~cet/facedatabase.html): Lisa DeBruine. Webmorph (Beta Release 2). Zenodo (2018). 10.5281/zenodo.1073696. Human face images not shown due to copyright restrictions.

Twenty images were used as visual stimuli, consisting of five different stimulus groupings, with four examples in each stimulus grouping (Fig. 1a). The five stimulus grouping were images depicting: human faces, scrambled human faces, pigeon faces, scrambled pigeon faces, and sine gratings of four different spatial frequencies. The human face images were obtained from the FEI face database available at (https://fei.edu.br/~cet/facedatabase.html). The images of pigeon faces were taken by the lead author (W.C.) using a Cannon DS126291 digital camera. Human face and pigeon face scrambled controls were created by dividing the face images into 15 × 32 square segments and then randomly shuffling the position and orientation of the tiles using open-source Webmorph software (https://webmorph.org/#P).

### Behavioural task

Pigeons were initially trained to respond with a single peck to a white dot to receive a grain reward. When pigeons were responding reliably to the white dot, they were then trained on a response inhibition task (Fig. 1b) during which they were required to withhold responses while a visual stimulus was displayed. Experimental sessions consisted of 160 trials, taking approximately 1 h to complete. Each image was presented 8 times, and all the stimuli were presented in a random order on each session. The procedure on a typical trial was as follows. At the end of a 6 s intertrial interval (ITI) period, a white dot was displayed in the centre of the response key during the ready period. Any pecks elicited during the pause period extended the pause period by 2 s. Two pecks to the white dot turned it off and initiated a pause period of a random time between 2 and 4 s. Any pecks in the first 0.5 s of the pause period were ignored to prevent pecks directed towards the ready stimulus from extending the duration of the pause period. Following the pause period, a stimulus period started during which an image belonging to one of the five stimulus groupings (human faces, scrambled human faces, pigeon faces, scrambled pigeon faces, or sine gratings) was displayed within the response window for a random duration between 1.5 to 3 s. Pecks during the stimulus period immediately turned off the stimulus and initiated a correction repeat of the same trial from the start of the ITI period. Following the stimulus period, a Go cue (grey square) appeared in place of the stimulus, letting the bird know that it was required to respond with a single peck to the Go cue. A peck to the Go cue turned it off and resulted in the start of the reward period with access to grain from the hopper for 1.75 s, accompanied by a 1000-Hz tone and the illumination of the hopper. To proceed to the next trial, the bird was required to peck the Go cue to deliver reward and initiate the ITI following the delivery of reward. Any pecks in the response window extended the ITI by 2 s.

### Surgery

**Figure 2:**
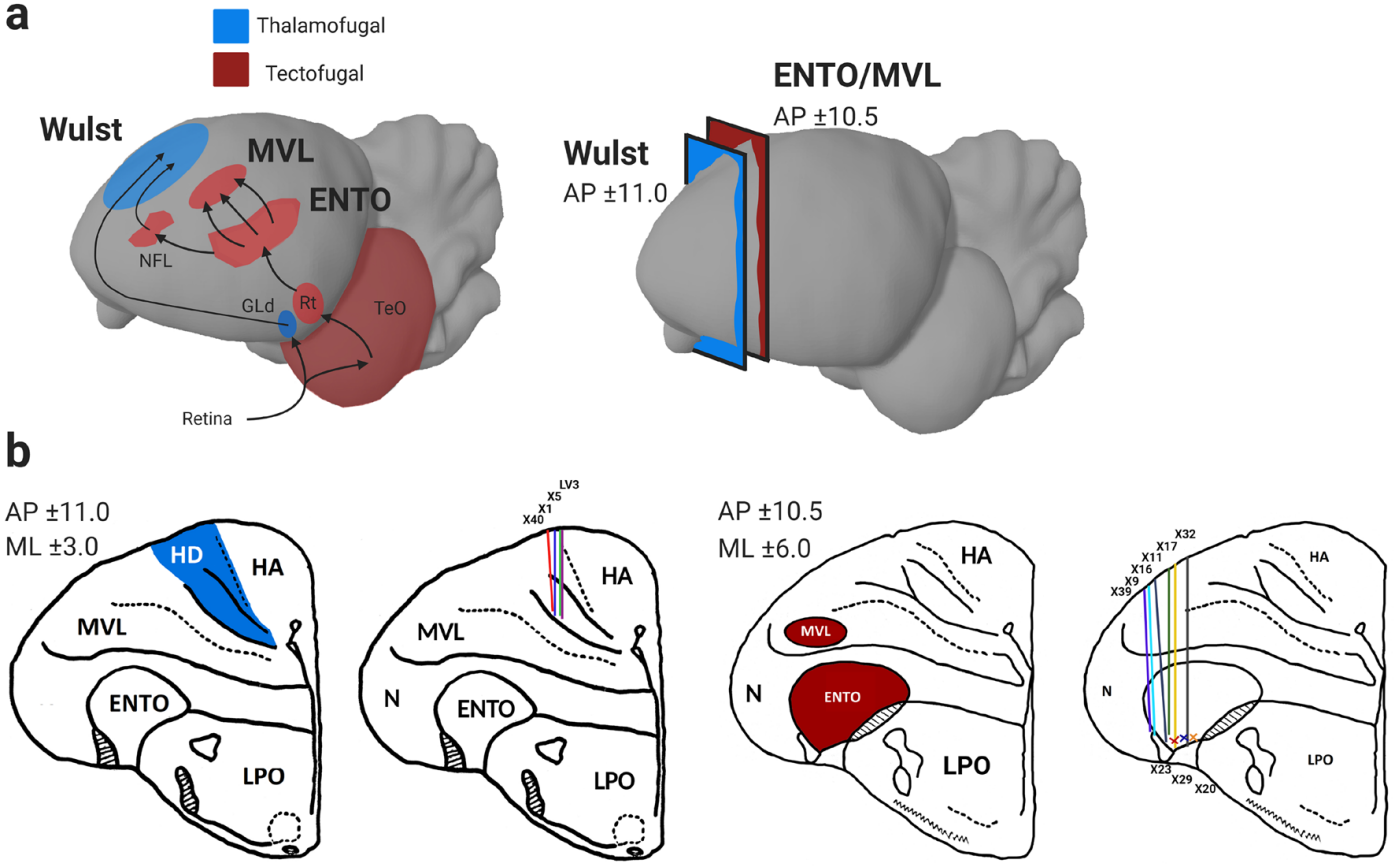
Targeted brain regions and electrode track records. (**a**) Sagittal depiction of the visual pathways of the pigeon brain (left) and coronal sections (right) corresponding to the locations of the targeted Wulst (blue) and ENTO/MVL (red) regions. Abbreviations^28^: optic tectum (TeO), nucleus rotundas (Rt), nucleus geniculatus pars dorsalis (GLd), nidopallium frontolaterale (NFL). (**b**) Visualisation of targeted MVL/ENTO (red) and Wulst (blue) regions and electrode track reconstruction for all pigeons (labelled with pigeon number) collapsed on the AP ± 10.5 and ± 11.0 coronal sections. Abbreviations^28^: Nidopallium (N), hyperpallium apiciale (HA), densocellular hyperpallium (HD), Lobus parolfactorius (LPO).

Once the pigeons were reliably completing the task, stereotaxic surgery was performed to install a movable microdrive into the target brain areas^26^. A mixture of Ketamine (30 mg/kg) and Xylazine (6 mg/kg) was injected into the pigeon’s legs as an anaesthetic. The feathers on the head were then removed. The pigeons were placed in a Revzin stereotaxic adapter^27^ to immobilise the head and a topical anaesthetic (10% Xylocaine) was applied to the scalp. The skin overlying the skull was retracted exposing the skull, and six stainless steel screws were inserted into the skull. One of these screws served as the ground screw. A hole was drilled above the targeted area and the dura was removed. A microdrive housing the electrodes was lowered into the hole until the tips of the electrodes were positioned above either MVL, ENTO, or Wulst (Fig. 2a). Ten pigeons (X9, X11, X16, X17, X20, X22, X23, X29, X32 and X39) had microdrives installed at positions AP ± 10.5 mm, and ML ± 6.0 mm, corresponding to the location of anterior MVL and ENTO. Four pigeons (X1, X5, X40, and LV3) had microdrives installed at positions AP ± 11.0 mm, and ML ± 6.0 mm, corresponding to the location of the Wulst. The microdrive was then secured to the skull using dental acrylic, and the wound was sutured closed. Xylocaine was applied again before the pigeons were placed into a padded and heated recovery cage. The pigeon remained in the recovery cage until it had returned to an active state, and was then returned to their home cage where they were given another 7 days to recover before experimental sessions began.

### Neuronal recording

The microdrives housed eight 25 μm Formvar-coated nichrome wires (California Fine Wire, Grover Beach, CA, USA) used to measure single neuron activity^26^. For each experimental session we searched for activity on any one of the eight wires and used one of the remaining wires as the indifferent. The signals were amplified using a Grass P511K amplifier (Grass Instruments, Quincy, MA, USA) and 50 Hz noise was eliminated using a notch filter. A CED (Cambridge Electronic Design, Cambridge, UK) electrophysiology system with Spike2 software stored and analyzed the data. Cells were isolated using CED’s template matching capacity (thereby eliminating artefacts) sampling at a rate of 20,000 Hz. The selection criterion was that the isolated neuron had a signal-to-noise ratio of no less than 2:1. A separate computer controlled the behavioural task and sent codes to the CED system to align key task events. Following each recording session, the electrodes were advanced approximately 40 μm before the pigeon was returned to their home cage. If we did not record from any neural activity the electrodes were moved approximately 20 μm, and the animal was returned to its cage. For the eight birds that were implanted in MVL, it was possible to subsequently record from ENTO due to its position directly ventral to MVL in the pigeon brain^27^. After advancing the electrode through the entire extent of MVL (2000 μm), the electrode was then advanced another 500 μm into ENTO, and subsequent recordings were performed through the extent of ENTO (3000 μm). For two birds (X9 and X29) we recorded directly from ENTO to balance the number of recorded neurons across MVL and ENTO. Recording sessions took approximately 1 h to complete. Pigeons completed one session daily for 5 days a week.

### Histology and electrode track reconstruction

At the end of the experiment, a 9 V potential was sent through each electrode for 10 s to create an electrolytic lesion marking the recording position of each electrode at the termination point in ENTO, MVL and Wulst. The pigeons were then euthanized using carbon dioxide gas, and were perfused with physiological saline and 10% formalin. The brains were removed from the skull and kept in 10% formalin for at least 5 days, followed by sucrose formalin (10% formalin, 30% sucrose). The brains were frozen and sliced into 40 µm sections and stained with thionin. Track reconstructions were made using the position of the electrolytic lesion and depth records. All electrode tracks were within the borders of the targeted ENTO, MVL and Wulst regions^27^ (Fig. 2b, and see Supplementary Table S1 for coordinates of electrode positions).

## Results

### Response dynamics of Wulst, ENTO and MVL single neurons

We analyzed neuronal responses on all 160 trials that the bird successfully inhibited responses to images until the grey square appeared, and discarded correction trials data from the analysis. To determine whether the neurons were visually responsive, each recorded neuron’s firing rates were first compared during 500 ms window post stimulus onset with a 500 ms window in the baseline ITI period using a paired *t*-test (p < 0.05). In Wulst we recorded from a total of 96 neurons of which 51 (53%) were visually responsive. In ENTO we recorded from a total of 140 neurons of which 88 (62%) were visually responsive. In MVL we recorded from a total of 120 neurons of which 77 (64%) were visually responsive. The proportion of neurons that were visually responsive was similar between the three regions (χ^2^ (2) = 3.18, *p* = 0.2).

**Figure 3:**
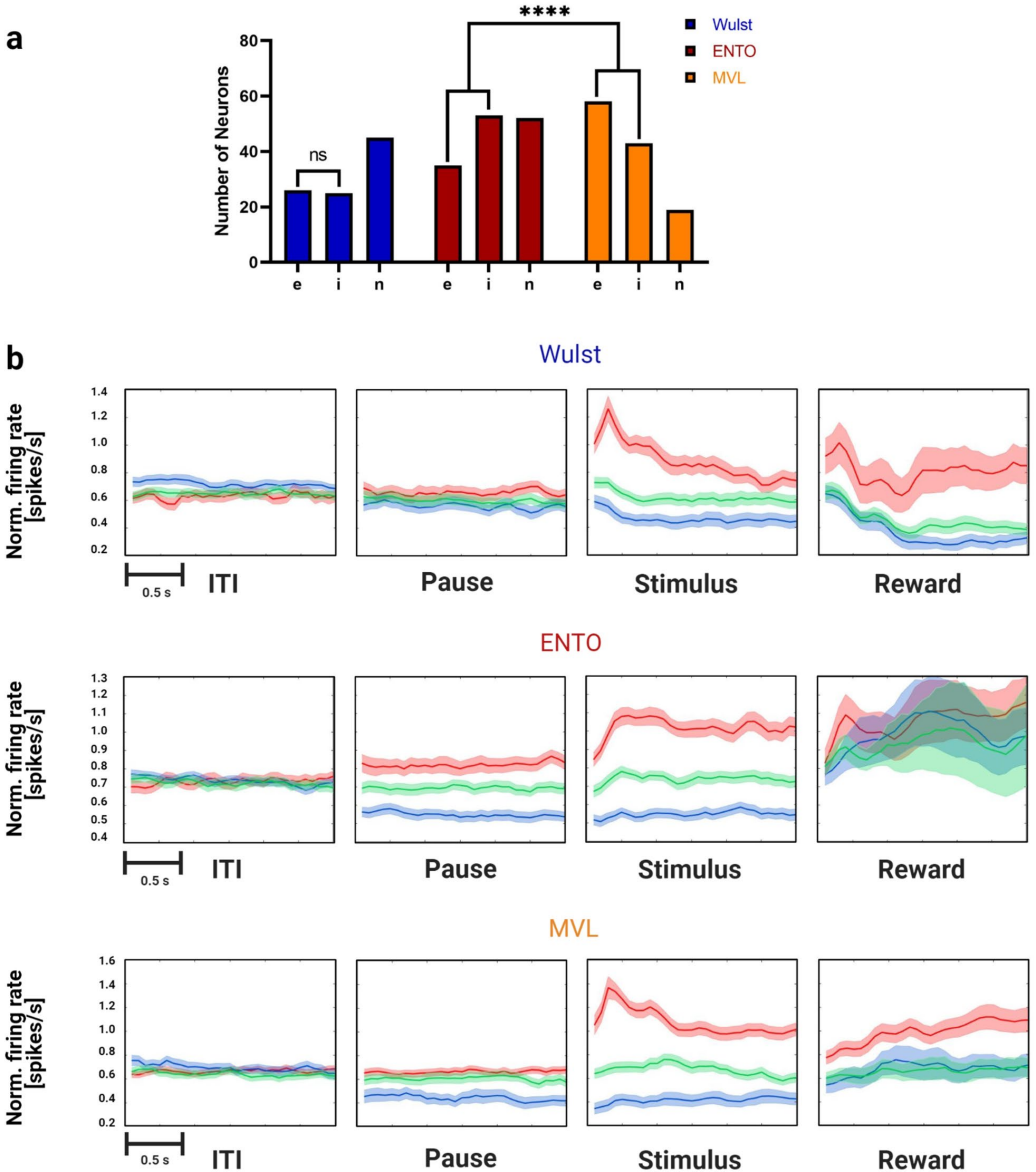
Single neuron response characteristics of Wulst, ENTO and MVL. (**a**) Totals of individual neurons that displayed excitatory (e) and inhibitory (i) responses to the visual stimuli, and totals that were non-responsive (n) to visual stimulation. Asterisks represent significant differences between regions for the relative proportions of visually-responsive/stimulus-selective neurons, and excitatory/inhibitory neurons: ****p < 0.00001. (**b**) Plots of normalised firing rate across each experimental period, calculated for excitatory (red), inhibitory (blue) and non-visually-responsive (green) neurons in Wulst, ENTO and MVL.

There were differences in the proportions of visually-responsive neurons that were excitatory and inhibitory between the three regions (Fig. 3a,b). Wulst displayed a similar proportion of 26 excitatory (51%) and 25 inhibitory (49%) neurons. In ENTO we found a greater proportion of 53 inhibitory (61%) compared with 35 excitatory (39%) neurons, whereas we found a greater proportion of 58 excitatory (75%) compared with 19 inhibitory (25%) neurons in MVL. There was a significant difference in the proportions of excitatory and inhibitory neurons between ENTO and MVL (χ^2^ (1) = 21.1, *p* = 0.00001), but not ENTO and Wulst (χ^2^ (1) = 1.64, *p* = 0.19).

The behavioural task required that the birds actively inhibited responses during the randomised pause period prior to visual stimuli appearing on the screen. Differences in neural activity between the three regions were observed during the pause period for the visually-responsive neurons (Fig. 3b). In Wulst, 26 of the 51 visually-responsive neurons (51%) also showed excitatory or inhibitory responses in the pause period. In ENTO, 57 of the 88 visually-responsive neurons (65%) responded in an excitatory or inhibitory manner during the pause period. In MVL, 34 of the 77 visually-responsive neurons (44%) responded with excitatory or inhibitory activity during the pause period. The relative proportion of visually-responsive neurons that became active during the pause period was substantially greater in ENTO relative to MVL (χ^2^ (1) = 7.05, *p* = 0.007), but not in ENTO relative to Wulst (χ^2^ (1) = 2.55, *p* = 0.11). The increased responsivity of ENTO during the pause period suggests that visually-responsive neurons may be modulated to a greater extent by attentional processes in anticipation of the upcoming visual stimulus in ENTO than at the level of MVL.

### Single-unit analysis of selectivity in Wulst, ENTO and MVL

**Figure 4:**
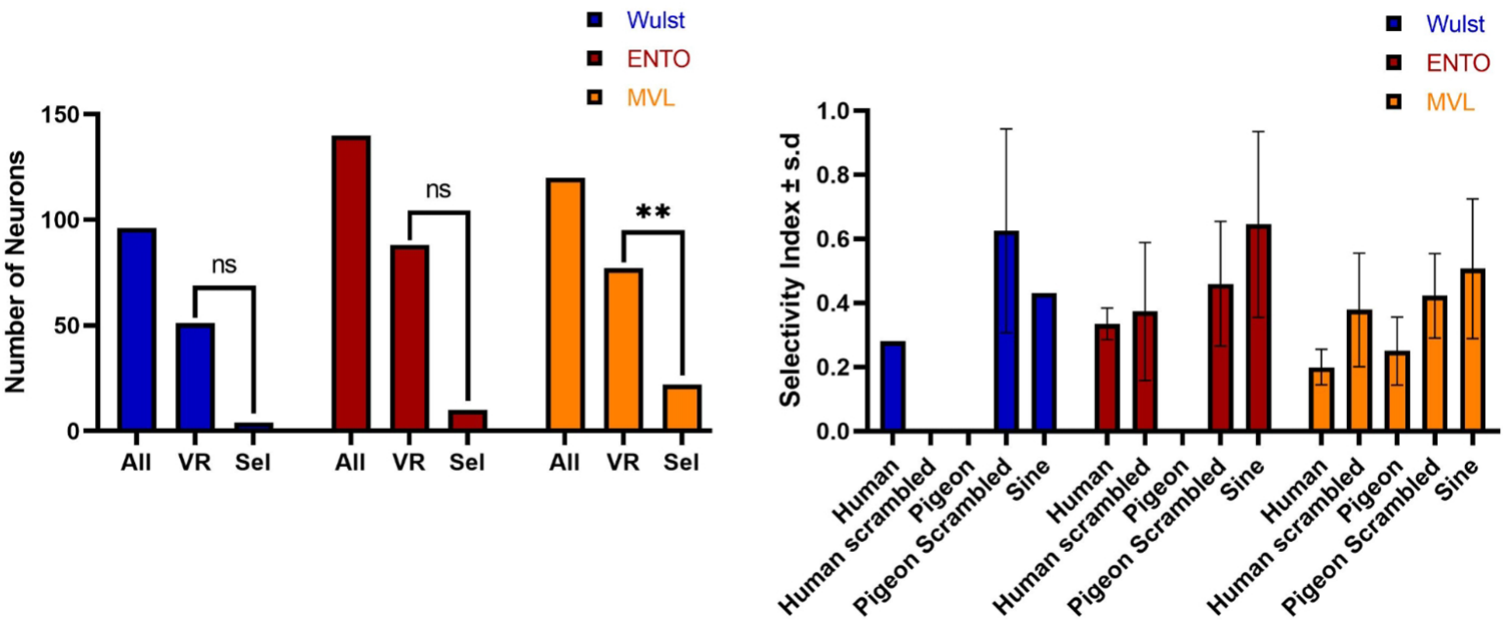
Stimulus selectivity of Wulst, ENTO, and MVL neurons. Comparisons between the three regions (left) of the total numbers of isolated neurons (All), visually-responsive neurons (VR), and stimulus-selective neurons (Sel): **p < 0.01. Average SI for stimulus-selective neurons in Wulst, ENTO and MVL (right). SI values reflect the degree to which a given group of neurons is selective for their preferred stimulus grouping. Greater SI values indicate larger differences between the average responses to the preferred stimulus grouping of a given neuron versus the averages of the other four stimulus groupings.

To determine if a visually-responsive neuron was sensitive to a particular grouping of stimuli, we compared the responses to each of the five stimulus groupings using a one-way AVOVA (*p* < 0.05). Neurons with a significant effect of stimulus grouping were further assessed using a Tukey Honest Significant Difference post-hoc comparison test (*p* < 0.05) in order to determine to which stimuli the neuron was responding, and a selectivity index (SI) was calculated to determine the magnitude of selectivity of the response (Fig. 4). The SI expresses the ratio of the average excitatory or inhibitory response to the preferred stimulus grouping of the neuron relative to the responses for the other stimulus groupings (see Supplementary Materials for single-unit data analysis). The classification system was previously used to map neuronal selectivity inside and outside of fMRI identified patches in macaque IT cortex^29^. Note that the classification of stimulus-selective neurons does not imply that a given neuron is exclusively “selective” for that particular grouping of stimuli, merely that of the five stimulus groupings tested, the preferred stimulus grouping produced the strongest response.

Of the 51 visually-responsive neurons in Wulst, 4 (8%) displayed a significant effect of stimulus grouping. The Wulst stimulus-selective neurons responded best to scrambled pigeon faces (n = 2: 50%) with a SI of 0.62 ± 0.22, human faces (n = 1: 25%) with a SI of 0.28, and sine gratings (n = 1: 25%) with a SI of 0.43. In ENTO, 10 of the 88 visually-responsive neurons (11%) displayed a significant effect of stimulus grouping. The ten ENTO stimulus-selective neurons responded best to sine gratings (n = 2: 20%) with an average SI of 0.64 ± 0.2, scrambled pigeon faces (n = 3: 30%) with an average SI of 0.46 ± 0.15, scrambled human faces (n = 3: 30%) with an average SI of 0.37 ± 0.17, and human faces (n = 2: 20%) with a SI of 0.33 ± 0.03.

**Figure 5:**
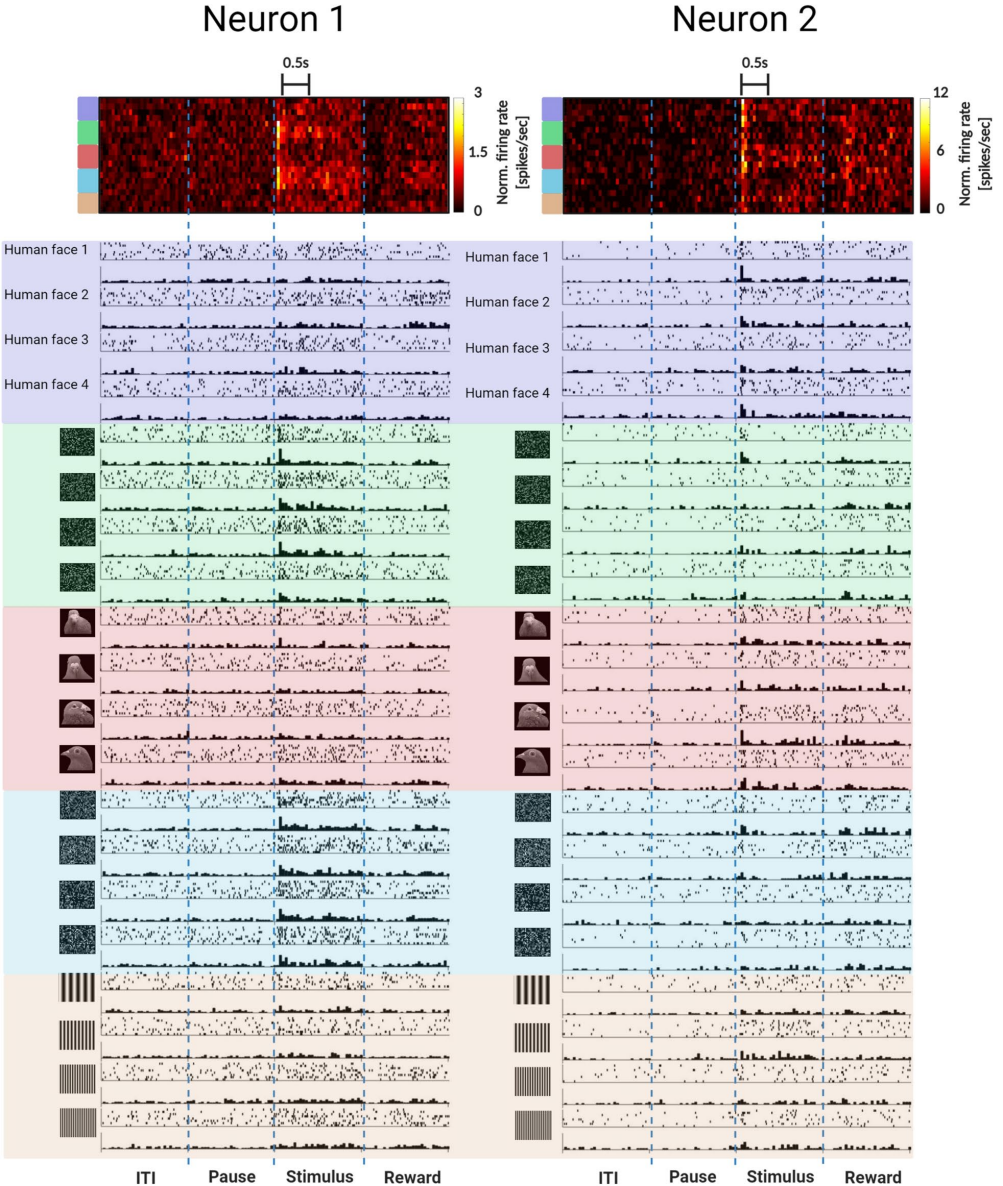
Examples of neurons that responded best to scrambled and face images. Two example MVL stimulus-selective neurons that responded best to scrambled human/pigeon faces (left) and pigeon/human faces (right). Scrambled images were created using open access Webmorph software (https://fei.edu.br/~cet/facedatabase.html): Lisa DeBruine. Webmorph (Beta Release 2). Zenodo (2018). 10.5281/zenodo.1073696. Human face images not shown due to copyright restrictions.

MVL displayed the greatest proportion of visually-responsive neurons sensitive to particular stimulus groupings, with 22 of the 77 visually-responsive neurons (29%) displaying a significant effect of stimulus grouping (Fig. 4). Three of the stimulus-selective MVL neurons responded best to sine gratings (n = 3: 14%) with an average SI of 0.5 ± 0.17. A greater number of MVL stimulus-selective neurons showed strong selectivity for scrambled pigeon faces (n = 5: 23%) with an average SI of 0.42 ± 0.11, and scrambled human faces (n = 6: 27%: see Fig. 5 for an example cell) with an average SI of 0.37 ± 0.16. Other MVL stimulus-selective neurons responded best to pigeon faces (n = 5: 22%: see Fig. 5 for an example cell) with an average SI of 0.25 ± 0.09, and human faces (n = 3: 14%) with an average SI of 0.2 ± 0.04. There were significant differences in the proportions of visually-responsive neurons in MVL that displayed a significant effect of stimulus grouping relative to ENTO (χ^2^ (1) = 7.77, *p* = 0.005) and Wulst (χ^2^ (1) = 8.14, *p* = 0.004).

Next, we verified that the number of neurons identified as stimulus selective in MVL was above expected chance level by generating simulated data of randomised firing rates for each stimulus grouping between the maximum and minimum values displayed by the real neurons during the stimulus period trials. We performed a one-way AVOVA (*p* < 0.05) comparing the responses between the five stimulus groupings for each of the 77 simulated visually-responsive neurons. Of the 77 simulated visually-responsive neurons, 4 (5%) displayed a significant effect of stimulus grouping, verifying that the high proportion of 22 out of 77 visually-responsive neurons (29%) that were stimulus selective was significantly greater relative to chance level (χ^2^ (1) = 14.99, *p* = 0.0001).

**Figure 6:**
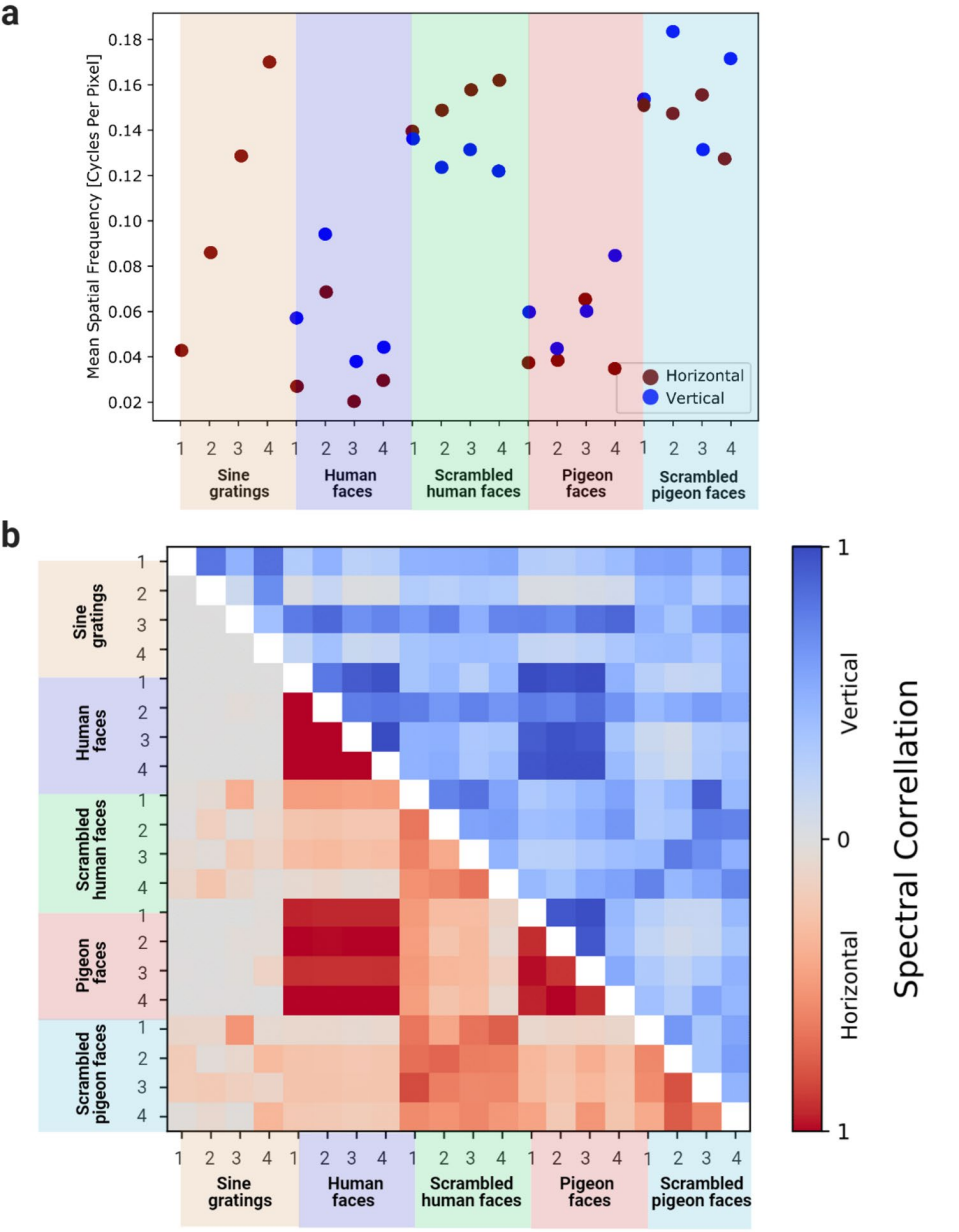
Spectral analysis of the image set. (**a**) The energy-weighted spectral average for a cross-section of each image in the horizontal and vertical directions, with the zero-frequency component omitted. The spatial frequencies of the scrambled images are centred much higher than for the unscrambled images, a result likely due to the high frequencies generated by the harmonics of the sudden transitions at the edges of the scrambled segments, and the interruption of low-frequency spatial information by the scrambling process. Vertical spatial frequency values for sine gratings are omitted for clarity as they only exist as an image compression artefact. (**b**) The energy-normalised spectral correlations between images, by a cross-section in the horizontal (upper triangle, red), and vertical (lower triangle, blue) directions.

Given that some neurons responded to the scrambled pictures, we next examined whether it was potentially the high spatial frequency information (corresponding to fine details and sharp edges/corners), or the low spatial frequency information (more global shape and broad swaths of luminance), to explain the selective responses of MVL neurons. To quantify the feature information of each stimulus grouping, we performed an analysis of the images’ Fourier amplitude spectrum (see Supplementary Materials). The spectral analysis showed that the grid-scrambling procedure resulted in greater high spatial frequency information for the scrambled images in comparison with the unscrambled images (Fig. 6a). Moreover, the scrambled images’ spectral information differed from the human faces and pigeon faces, which were highly correlated for low spatial frequencies (Fig. 6b). While we did not match the images’ luminance before performing image scrambling, cells responsive to scrambled images didn’t also respond to the unscrambled human and pigeon versions that shared the same luminance, and vice versa. The selective responses to scrambled or face images are therefore attributable to the differences in the features of the images, rather than differences in luminance.

### Population-level analysis of selectivity in Wulst, ENTO and MVL

Our single-unit findings indicated that MVL displayed a greater proportion of stimulus-selective neurons than ENTO and Wulst. That said, it is clear that coding object information is achieved mainly by a population of neurons^2, 20, 30^. We therefore next examined whether at the population level, MVL also discriminated better among the stimulus groupings. We evaluated the stimulus-discrimination capacity of all the visually-responsive neurons sampled from each region using a LDA with permutation resampling (see Supplementary Materials for population data analysis). As each neuron was recorded on sequential days and one cannot associate the responses of the single-trial firing rates to form true multivariate observations, the LDA procedure was performed 1200 times while shuffling the data lists before associating vectors with stimulus grouping labels (permutation resampling), to generate different sets of vectors from the same data. Others have used LDA with permutation resampling to extract information from single-trail responses to objects in monkeys^30^.

**Figure 7:**
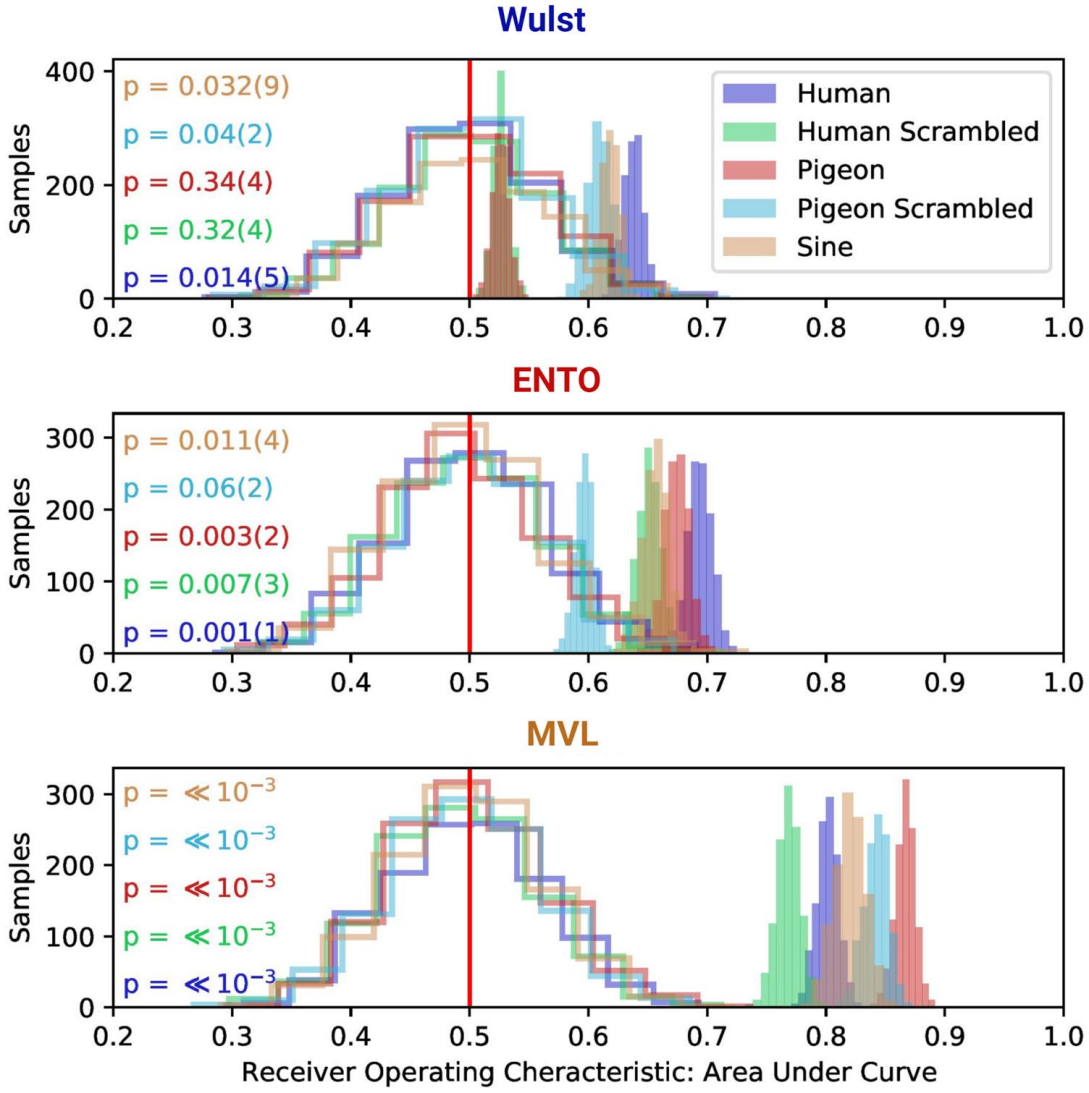
LDA performance shows that the MVL population response encodes stimulus feature information. The un-filled distributions show the performance of the LDA trained on randomly labelled data, which contain "no information" for the permutation significance test. The shaded distributions show the performance of the correctly labelled data. p-values (and their error) are shown to the left for each stimulus grouping (color coded on the left). The p-value for each stimulus grouping is derived from how far away from the "no information" distribution of samples that the correctly labelled performance falls.

For the Wulst population, the distribution of the receiver operating characteristic (ROC) for each stimulus grouping did not deviate significantly from chance performance under the null hypothesis distribution (Fig. 7), indicating that very little stimulus feature information was accessible from the population code of the thalamofugal visual pathway. The population response of ENTO distinguished between most of the stimulus groupings with classification performance higher than > 99% of the samples of the estimated null hypothesis distribution, with the exception of scrambled pigeon faces with > 90% of the samples higher than the null hypothesis distribution (Fig. 7). The MVL population response, however, discriminated between all of the stimulus groupings tested with > 99% classification performance compared with the null hypothesis distribution, displaying greater stimulus feature information than the Wulst and ENTO populations (Fig. 7). We verified that the population responses of the LDA classifier for the three regions generalised to held-out images for each stimulus grouping (see Fig. 1 in Supplementary Materials).

We also compared the average response to the original images versus the scrambled images for all of the visually-responsive neurons from each region to determine whether they displayed a preference for the scrambled images over the originals. There were no significant differences in the average responses for the scrambled images (mean: 6.88 spikes/s) compared with the original face images (mean: 6.39 spikes/s), (paired *t*-test, *t*(76) = 5.56, *p* = 0.07) for the MVL visually responsive neurons. There were also no significant differences between the average responses to the scrambled images (mean: 9.01 spikes/s) compared with the original images (mean: 9.14 spikes/s) for the 88 visually-responsive ENTO neurons (paired *t*-test, *t*(87) = 1.5, *p* = 0.39). Likewise, there were no significant differences between the population average responses to the scrambled images (mean: 4.68 spikes/s) compared with the original images (mean: 4.77 spikes/s) for the 51 visually-responsive Wulst neurons (paired *t*-test, *t*(50) = − 0.95, *p* = 0.52).

## Discussion

We investigated the response selectivity of three visual forebrain areas of the pigeon brain at the single cell and population level, and determined the coding principles of these regions, with a special emphasis on face perception. We found that the pigeon MVL displays a greater proportion of stimulus-selective neurons than ENTO and Wulst. None of the stimulus-selective neurons identified were truly face-selective, consistent with past studies of the pigeon^25, 31^ and crow^32^ visual system.

Our finding of single neurons responsive to scrambled images in MVL was likely due to the overall increase in power across all spatial frequencies when compared with the original images. Sensitivity to additional power introduced by image scrambling is well documented in mammals, where it is mostly observed in early visual cortex of macaque monkeys^33^, rats^34^, and also the early layers of computational models of object recognition^35^. ENTO and MVL are part of separate layers in the DVR, with ENTO receiving its primary sensory input from the thalamus^15^. Since both the mesopallium and nidopallium layers are both heavily reciprocally connected with ENTO^13, 15^, MVL is a natural candidate for displaying sensitivity to more complex visual form than at the level of ENTO. We observed in the present study that while the numbers of stimulus selective cells is greater in MVL relative to ENTO, the selectivity may be driven by low-level image features, as would be expected in early visual cortex.

Beyond the single-unit analysis, the population response of the visually-responsive neurons sampled from the three regions indicates that information associated with stimulus features is recoded in a more readable form between ENTO and MVL. A recent study using LDA and adopting the same stimulus set used in studies of macaque^2^ and human^36^ IT cortex showed that MVL responses distinguished between the features of animate and inanimate object categories with greater accuracy than an ENTO population^20^. The differences in population responses that we observed between ENTO and MVL suggests that neurons in separate layers of the DVR display different sensitivity to visual form within the avian canonical circuitry. However, these differences are not consistent with a progression in selectivity for objects over scrambled stimuli equivalent to that observed between early visual and extrastriate cortex. Consistent with divergent strategies for object recognition in birds compared with primates, pigeons more readily attend to the local details of stimuli rather than their global configuration^37, 38^. For example, pigeons strongly rely on the high spatial frequency components of images viewed in the frontal visual field as the most diagnostic level of information during picture memorization^39^. While it is possible to train pigeons to report information at the global scale by directing attention to features shared across images belonging to the same category^40, 41^, they are predisposed to attend to stimuli at the local level.

Some of the stimulus-selective MVL neurons did also respond best to images of faces. The average selectivity index values for faces displayed by MVL neurons, however, are lower than the minimum selectivity index value of 0.33 (corresponding to a 2:1 ratio of face-to-non-face category response) required for classification of face-selective neurons in macaque face-patches (average SI for faces of 0.87)^42^. It is possible that the lack of strong selectivity for faces is related to fundamental differences between birds and primates in holistic face processing^43^. Unlike humans and non-human primates, pigeons’ memory performance for images of primate faces is unimpaired by inversion of the faces^44^, and discriminations between faces are based primarily on an additive integration of local features^45^. The absence of strong selectivity displayed to the human and pigeon faces suggests that pigeons do not possess circuitry dedicated to face-perception analogous to the face-patch system, and these selective responses may reflect sensitivity to the general similarity in low spatial frequency content shared across the natural images depicting faces. It is also important to note that future studies with the aim of assessing face-selectivity in the avian brain will need to also include images of non-face objects to disentangle face-selective responses from a general selectivity for natural images over scrambled stimuli.

Understanding how information is processed among the different visual regions of the avian brain is still very much in its infancy. On the basis of the findings from previous studies and the current study, we tentatively propose that object categorisation in the pigeon brain may not depend on a stage of holistic/viewpoint-invariant representation comparable to higher stages of the primate ventral stream. We cannot rule out the possibility that the visual nidopallium^16^ and the associative nidopallium caudolaterale (the avian equivalent of prefrontal cortex^46, 47^) may be involved in categorical representation of objects. It is also possible that the thalamofugal pathway integrates representations of object features at a more global spatial scale when presented laterally at distance in comparison with the tectofugal pathway. The low number of stimulus-selective neurons we found in Wulst may be because the thalamofugal pathway participates mainly in lateral object vision^8–10^. As the Wulst also displays small receptive field sizes^6, 7^ future studies in freely moving pigeons could use search stimuli^48^ to map the receptive fields of neurons so that image size and position in the visual field is adjusted according to a given neuron’s preference.

In summary, we found evidence that MVL displays greater selectivity to visual stimuli in comparison with ENTO. In comparison with the tectofugal pathway, the Wulst of the thalamofugal pathway is less involved in object feature analysis in the frontal visual field, and is likely to be specialised for lateral object vision. Further electrophysiological studies are required to determine how the transformation of information between different layers of the sensory DVR and Wulst constructs representations of objects in the avian brain.

## Supplementary Information

Supplementary Figure 1.

Supplementary Information.

## Data Availability

All data that support the findings of this study are available from the corresponding author upon reasonable request.

## Code availability

The analysis code supporting the findings of this study are available from the corresponding author upon reasonable request.
