# Supplementary figures and images for: Neurons in the pigeon visual network discriminate between faces, scrambled faces, and sine grating images
Source: Sci Rep. 2022 Jan 12;12:589. doi: 10.1038/s41598-021-04559-z (PMC8755821; doi:10.1038/s41598-021-04559-z)

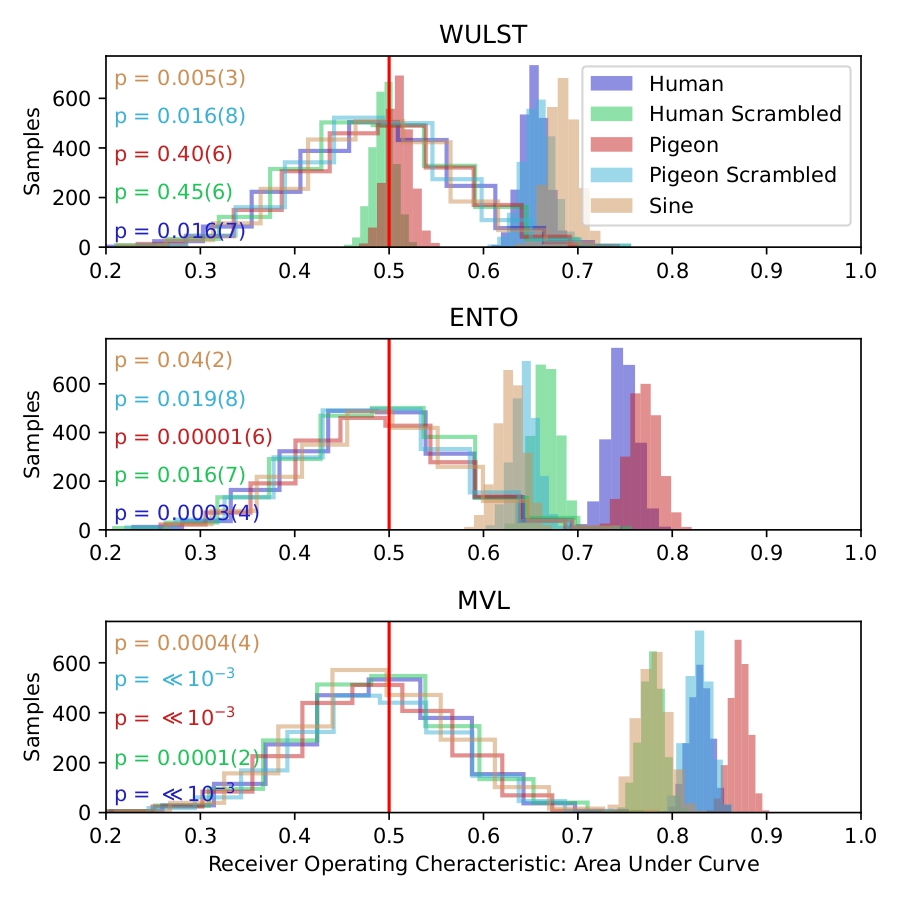

Supplement: Supplementary file 1 — Supplementary Figure 1. [file 41598_2021_4559_MOESM1_ESM.jpg]
