# Supplementary material for: Neurons in the pigeon visual network discriminate between faces, scrambled faces, and sine grating images
Source: Sci Rep. 2022 Jan 12;12:589. doi: 10.1038/s41598-021-04559-z (PMC8755821; doi:10.1038/s41598-021-04559-z)
Supplement: Supplementary file 2 — Supplementary Information. [file 41598_2021_4559_MOESM2_ESM.docx]

**Supplementary Materials**

**Histology**

The intended and actual AP and ML electrode track placements for each bird is shown in Supplementary Table 1.

**Supplementary Table 1.** Intended and actual electrode track Anterior/Posterior (AP) and Medial/Lateral (ML) positions for each bird. Asterisks denote that the electrode tracks were not visible but the termination point was recovered based on the visible location of the craniotomy during blocking and the electrode depth records.

| **Subject ID** | **Intended AP (mm)** | **Actual AP (mm)** | **Deviation AP (mm)** | **Intended ML (mm)** | **Actual ML (mm)** | **Deviation ML (mm)** |
| --- | --- | --- | --- | --- | --- | --- |
| LV3 | ± 11.0 | ± 11.0 | 0.0 | ± 3.0 | ± 2.7 | -0.3 |
| X1 | ± 11.0 | ± 10.75 | -0.25 | ± 3.0 | ± 3.2 | +0.2 |
| X5 | ± 11.0 | ± 11.0 | 0.0 | ± 3.0 | ± 3.0 | 0.0 |
| X9 | ± 10.5 | ± 10.25 | -0.25 | ± 6.0 | ± 7.1 | +1.1 |
| X11 | ± 10.5 | ± 10.75 | +0.25 | ± 6.0 | ± 6.7 | +0.7 |
| X16 | ± 10.5 | ± 9.5 | -1.0 | ± 6.0 | ± 6.9 | +0.9 |
| X17 | ± 10.5 | ± 11.0 | +0.5 | ± 6.0 | ± 6.3 | +0.3 |
| X20 | ± 10.5 | ± 9.5 | -1.0 | ± 6.0 | ± 6.1 | +0.1 |
| X22 | ± 10.5 | ± 11.0 | +0.5 | ± 6.0 | ± 6.2 | +0.2 |
| X23 | ± 10.5***** | ± 10.5***** | 0.0***** | ± 6.0***** | ± 6.0***** | 0.0***** |
| X29 | ± 10.5***** | ± 10.5***** | +0.0***** | ± 6.0***** | ± 6.4***** | +0.4***** |
| X32 | ± 10.5 | ± 10.5 | 0.0 | ± 6.0 | ± 5.8 | -0.2 |
| X40 | ± 11.0 | ± 10.75 | -0.25 | ± 3.0 | ± 3.4 | +0.4 |
| X39 | ± 10.5 | ± 11.25 | +0.75 | ± 6.0 | ± 7.2 | +1.2 |

**Single neuron analysis**

Each neuron’s spiking data for behavioural sessions was loaded into MATLAB (version R2016B) for data analysis. Neurons were required to exhibit baseline mean firing rates > 0.2 Hz during the ITI period (middle 500 ms of the ITI) to be included in the analysis. Neurons were classified as visually responsive if the firing rate over all 160 trials in the stimulus period (500 ms post stimulus onset) was significantly greater (paired *t*-test, two tailed, p < 0.05) than the neurons firing rate during the baseline ITI period. The response was defined as ‘excitatory’ if the average response in the stimulus period was greater than the average baseline ITI response. The response was classified as ‘inhibitory’ if the average response in the stimulus period was less than the average baseline ITI response.

To determine if a visually responsive neuron was sensitive to a particular stimulus grouping, we compared the responses to each of the stimuli in the 5 stimulus groupings using a one-way AVOVA Neurons with a main effect of stimulus grouping (*p* < 0.05) were defined as “stimulus-selective”. Neurons with a significant (p < 0.05) effect of stimulus grouping were further assessed to determine which stimulus grouping were driving the effect (Tukey-HSD post-hoc comparison test, p < 0.05). If the neuron was excitatory the stimulus groupings with significantly greater firing rates were used to determine the neurons selectivity, whereas if the neuron was inhibitory the stimulus groupings with significantly lower firing rates were used to determine the neurons selectivity. The degree to which a neuron was selective for a given stimulus grouping was calculated using the following formula:

$\mathrm{SI}_{a}=\frac{R_{a}-1/4\left( R_{b}+ R_{c}+ R_{d}+R_{e} \right)}{R_{a}+1/4(R_{b}+ R_{c}+ R_{d}+R_{e})}$(1)

Where SI_a_ is the selectivity index for stimulus grouping a, R_a_ is the response to stimulus grouping a, and R_b_–R_e_ are the responses to the remaining stimulus groupings. We subtracted the average baseline ITI response from the average responses to each stimulus grouping before calculating SI values to account for differences in cells baseline activity. A high SI value indicates a stimulus grouping response that is very different from the average response to the remaining four stimulus groupings (i.e., strong selectivity); a low absolute SI value indicates a stimulus grouping response that is only marginally different from the average response to the remaining four stimulus groupings (i.e., weak selectivity).

**Spectral data analysis**

Image data is a 2D structure, and while the data itself is easy to visualise, it is difficult to identify features in 2D spectrographs except for a subset of cases with carefully chosen scaling. 1D spectrums are much easier to identify the features in images. The question then becomes how to go about reducing the 2D image to 1D, which naturally incurs some loss of information. One approach is to average the images entire spectrum in a particular direction e.g., horizontal/vertical dimensions. A disadvantage of averaging is the “blurring" of the spectrum which will always result in some information loss.

To compare the feature content of the images, we calculated the energy-weighted spectral average for a cross-section in the horizontal and vertical directions. Taking a cross-section results in a very sharp distribution of pixels when compared to averaging over the entire image in a particular direction, making it slightly easier to identify features. The spectrum of a cross-section was determined by taking the power spectral density of a row or column of pixels from the centre of the image for the horizontal and vertical directions. Qualitatively, the results are equivalent to those obtained using spectra averaged over the entire image.

To reduce the 2-D spectrum to a 1D spectrum, we averaged the spectrum of the cross-section, ignoring the zero-frequency component. This is equivalent to subtracting out the average brightness of the image (the zero-frequency component is the total brightness of the image, and dominates the average if left in), or “blocking the DC component”. From the power spectrum $P_{n}(f)$ which we know for discrete frequencies $f_{i}$, we next extracted the spatial frequency. This is equivilent to taking the average spatial frequency weighted by the spectral power of the image:

$f_{\mathrm{avg}}=\frac{\sum_{i,\nu_{i}\neq0} P_{n}(f)f_{i}}{\sum_{i,\nu_{i}\neq0} P_{n}(f_{i})},$ (2)

Correlations were generated from the power spectrum $P_{n}(f)$ for the $n$-th image, by the energy normalised cross-correllation:

$X_{nm}=\frac{\sum_{i,f_{i}\neq0} P_{n}(f_{i})P_{m}(f_{i})}{\left( \sum_{i,f_{i}\neq0} P_{n}(f)^{2} \right)\left( \sum_{i,f_{i}\neq0} P_{m}(f_{i})^{2} \right)}$ (3)

Alternatively, the normalised power spectrum can be defined as:

$p(\nu_{i})=\frac{P(f_{i})}{\sum_{j,f_{j}\neq0} P(f_{j})^{2}}$ , (4)

and then the correlation is the sum of the product:

$X_{nm}=\sum_{i,\nu_{i}\neq0} p_{n}(f_{i})p_{m}(f_{i})$ (5)

That is, the average spatial frequency weighted by the spectral power of the image.

**Linear discriminant analysis (LDA) with permutation re-sampling**

Detection of the population code in each visual region required a comparison of the neural activity patterns across different stimulus conditions. Individual neurons’ firing rates in the stimulus period across the eight presentations of each stimulus were collated into a population vector$\mathbf{X}$. As each stimulus was repeatedly presented, the variability of the responses produced a cluster of points in the high dimensional space of the neuronal population.

As each neuron was recorded on sequential days, there is no unique association of presentations to produce each**.** We eliminated this issue by performing a resampling procedure, separately permuting the firing rates of each neuron for each stimulus. Each sample produces a different set of population vectors from the same data.

We determined whether the data points for each stimulus in the space can be divided into distinct regions (classified) based on which stimulus grouping the points belong to. We used a linear discriminant analysis (LDA) to identify the linear boundaries between data points belonging to different stimulus groupings. We trained the LDA classifier on each stimulus grouping of interests data (labelled in-class), and tested the classification performance compared with the other four stimulus groupings data (labelled out-class).

The boundaries between stimulus groupings in the LDA was calculated as:

$g\left( \boldsymbol{x} \right)={\boldsymbol{W}^{t}}_{x}+ \theta$ (6)

Where $\boldsymbol{W}$ defines the direction of the linear boundary and its offset (θ). Each population vector belongs to the training stimulus grouping if g(x) ≥ 0. The LDA determines the linear function that determines the maximum ratio of within-class scatter vs. between-class scatter on the training data:

$J\left( \boldsymbol{W} \right)= \frac{{|\tilde{m}_{1}-\tilde{m}_{2}|}^{2}}{\tilde{s}_{1}^{2}+\tilde{s}_{1}^{2}}$ (7)

Here, $\tilde{m}_{i}$ is the mean for the projected data points for the sample *i*, and $\tilde{s}_{1}^{2}=\sum_{y\in y_{i}} (y-{\tilde{m}_{1})}^{2}$is the scatter for projected sample *i*. The vector **W** that maximises J in equation 7 is:

$\boldsymbol{W}=S_{W^{-1}}$($\tilde{m}_{1}-\tilde{m}_{2}$) (8)

Here, $\boldsymbol{S}_{\boldsymbol{W}} = \sum_{i=1}^{2} \sum_{x\in D_{i}} {(x- m_{i})(x- m_{i})}^{T}$ is the scatter matrix. The solution for the threshold is:

$\theta= \frac{1}{2} ( m_{1}+ m_{2})(x- m_{i})$ (9)

The LDA classifier learned to distinguish between two stimulus groupings at a time. Two thirds of the population vectors were randomly assigned to the training data, and the remaining population vectors were assigned to the test data, with the test-train cycle procedure repeated three times so each third of the data was used as the test data ($K$-folds cross-validation with $K$=3). We labelled the stimuli in the training data as in-class or out-class separately for each stimulus grouping, trained a LDA on the data, and then calculated the linear boundary between population vectors in the test data using the scikit-learn package in Python. Test performance was evaluated separately for each stimulus grouping and quantified as the area under the curve (AUC). As the classification performance depends on the random assignment of population vectors to training or test data, the procedure was repeated 1000 times. To determine the statistical significance of the classifier performance, we computed the classification score on permuted data, where the class labels of the images in the test sets were randomly shuffled using a Kolmogorov–Smirnov test. As no stimulus grouping information was present in the test set for the permutation test, the classifier should result in chance performance of 50%. In contrast, if the population code represents stimulus information, the classifier performance on the recorded data with correct class labels should be significantly better than the performance on the permuted data.

**LDA with leave one group out (LOGO)**

We ran the LDA procedure for 1000 iterations using LOGO instead of k-folds cross validation to determine weather the classifier could generalise to the remaining image for each stimulus grouping it hadn’t been trained on (see Supplementary Figure 1 below). By performing LOGO as a comparison to using K-folds cross validation, we were able to assess whether the population firing rates did truly display selectivity to a particular stimulus grouping (e.g., face-selectivity), as opposed to displaying selectivity to only the four images in the dataset that had been part of the train-test cycle. The LDA with LOGO procedure differed from the k-folds procedure only in that two thirds of the population vectors were randomly assigned to the training data, and the vectors corresponding to the held-out image were assigned to the test data.


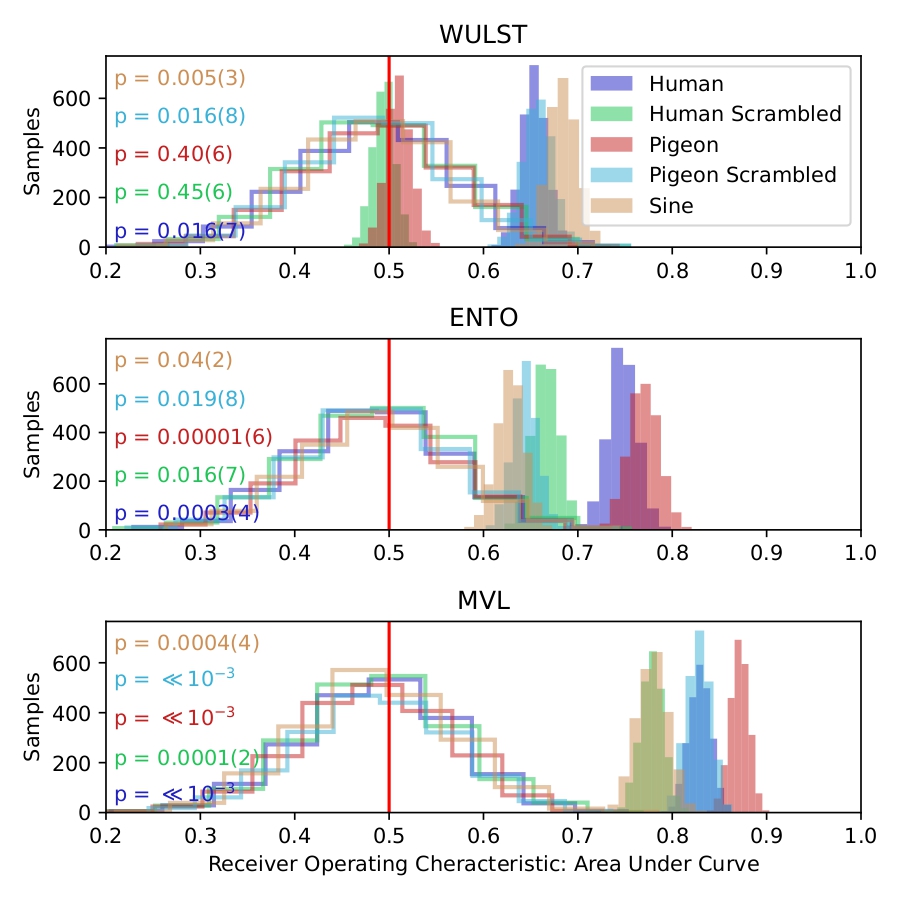


**Supplementary Figure 1. LDA performance using LOGO instead of K-folds cross validation.** The un-filled distributions show the performance of the LDA trained on randomly labelled data, which contain "no information" for the permutation significance test. The shaded distributions show the performance of the correctly labelled data. P-values (and their error) are shown to the left for each stimulus grouping (color coded on the left). The p-value for each stimulus grouping is derived from how far away from the "no information" distribution the correctly labelled performance falls.

We found that there we no significant differences between the LDA classifier when run using k-folds (see Figure 7 in main text) and LOGO procedures, verifying that the population response to each stimulus grouping generalised well to the left out images that were not part of the train-test cycle.
